# Allergy diagnosis in Geneva area: a complex multi-ethnic community with high pan-allergen prevalence

**DOI:** 10.1186/2045-7022-4-S2-P49

**Published:** 2014-03-17

**Authors:** Fabienne Gay-Crosier, Domingo Barber, Jacques Bienvenu

**Affiliations:** 1 Clinical Allergy and Clinical Immunology, Internal Medicine FMH, Carouge, Geneva, Switzerland; 2 Universidad San Pablo CEU, IMMA-Facultad de Medicina, Madrid, Spain; 3 Centre hospitalier Lyon-Sud, Laboratoire d'immunologie, Lyon, France

## Background

Epidemiological studies are of great importance to understand the relevant allergens for a given population. Geneva is situated in Central Europe, and patients attending to allergy office are of diverse ethnic origin. We aimed to analyze sensitization profiles and evaluate a previously perceived high prevalence of pan-allergen sensitization in our population.

## Methods

Seventy patients with seasonal allergy symptoms were consecutively included over a one month period following clinical practice and classified in three groups: Western / Central / East Europe (30), South Europe (21), Asia / Africa / South America (19). Routine SPT panel including SPT of purified palm tree pollen profilin, ISAC-CAP microarray and a careful clinical evaluation was performed.

## Results

13/30, 13/21 and 6/19 were sensitized to profilin, while 14/30, 11/21 and 5/19 were positive to Bet v 1. 40% of profilin positive patients had clinical food allergy with melon or water melon. ISAC profilin results were compared at different cut-off levels with SPT to pure profilin. 11 patients were serologically profilin positive between 0.01 and 0.29 ISU ; 7 of them were SPT profilin positive and 4 of them had oral symptoms induced by melon.

## Discussion

In Areas with a high pan-allergen sensitization prevalence, the use of CRD is unavoidable. Multiplexed platforms like ISAC CAP arrays have become for us a routine diagnosis tool. All studied groups showed a high prevalence of profilin and PR10 resulting in complex food related allergy. The analysis of the ISAC results with the recommended current cut-off values might not be adequate. Profilin is present in all vegetables, and thus is ingested or inhaled in a daily-seasonal basis. Perhaps the relative high sIgG4 to profilin can interfere ISAC result. As described previously, profilin seems to be connected to grass allergy. All patients but one in this study were grass sensitized (Phl p 1 and or Phl p 5 positive). Grass pollen counts in Geneva are in the range of 100 to 1000 grains/m3. A similar grass exposure related to high profilin prevalence has been described in western Spain, making us think that this is not an isolated phenomenon and that exposure and cofactors conditions are very region dependent. Taking all this into account confirms the absolute need for a suitable use of micro-arrays technology in daily practice in order to be able to perform individually adapted successful treatments.

